# Rapid Adaptation to Prevent Drug Use (RAPD): protocol of a pilot randomized trial to enhance the impact of an evidence-based intervention for youth

**DOI:** 10.1186/s40814-024-01581-6

**Published:** 2025-01-21

**Authors:** Andria B. Eisman, Christine Koffkey, Robert T. Partridge, Suzanne Brown, Bo Kim

**Affiliations:** 1 Division of Kinesiology, Health and Sport Studies, College of Education, Faculty/Administration Building, Community Health, Wayne State University, Detroit, MI 48202 USA; 2 Research Design and Analysis Unit, Department of Psychology, Wayne State University, Detroit, MI 48202 USA; 3 School of Social Work, Wayne State University, Detroit, MI 48202 USA; 4 Center for Healthcare Organization and Implementation Research, VA , Boston Healthcare System, 150 South Huntington Avenue, Boston, 02130 USA; 5 Department of Psychiatry, Harvard Medical School, 25 Shattuck Street, Boston, MA 02115 USA

**Keywords:** Behavioral intervention, Economics, Adolescence, Drug use, Education, Implementation science, Prevention

## Abstract

**Background:**

Drug use trends change rapidly among youth, leaving intervention experts struggling to respond promptly. Delays in responses can lead to preventable morbidity and mortality. The COVID-19 pandemic underscored the need for implementation science to facilitate rapid, equitable responses using existing treatment and prevention efforts. Existing, widely adopted evidence-based interventions (EBIs; e.g., the Michigan Model for Health™: MMH) are well suited to address emerging drug trends. We have a *critical need* to advance implementation strategies to optimize system responsiveness to these emerging drug issues. This research aims to design and test implementation strategies to (1) improve the responsiveness of school-based EBIs in addressing urgent issues and (2) find ways to support teachers in implementing updated EBIs, attending to unique considerations of schools serving economically disadvantaged students.

**Methods:**

The research aims are as follows: aim 1: identify implementation gaps and best practices using After Action Review (a reflective process used by health organizations in responding to emergent public health events) using qualitative methods. Aim 2: design and pilot test RAPD (Rapid Adaptation to Prevent Drug use) based on aim 1 findings. RAPD refers to a novel set of implementation strategies designed to enhance the capacity of an existing, widely adopted evidence-based universal prevention curriculum (MMH) to respond to emerging drug issues among youth. We will pilot test RAPD in ten middle schools serving diverse student populations using a two-group, mixed method, cluster randomized controlled trial design. Aim 3: assess the costs and benefits of RAPD from multiple partner perspectives using a mixed methods approach.

**Discussion:**

This study focuses on designing and deploying implementation strategies to reduce the detrimental impact of emerging drugs and provide an infrastructure to make future adaptations that can be applied in other contexts. After Action Review (AAR) provides a valuable opportunity to review the statewide response to past drug use events, specifically the vaping crisis, using the MMH curriculum, which can systematically guide implementation strategy selection and deployment to meet identified gaps. The rationale for the proposed research is that designing and testing RAPD will advance implementation science in responding to urgent public health events and ensure equitable responses across youth populations.

**Trial registration:**

ClinicalTrials.gov NCT05806840.

## Background

Drug use trends change rapidly among adolescents [1, 2], yet schools and other organizations engaged in prevention efforts struggle to respond quickly and effectively to these changing trends. Stemming the onset and rapid escalation of drug use requires swift responses. Vaping among youth is one example of an urgent public health problem that has escalated rapidly. Rates of vaping increased by 51% among high school students and 45% among middle school students from 2017–2019 [1]. The response happens in schools for many public health issues that affect youth [3]. Schools are ideal settings for prevention because they are critical contexts that shape health behaviors and offer one way to increase access to services for low-income youth [4–6]. Developing and testing new interventions for each emerging public health issue, however, is time- and resource-intensive, unlikely to fit schools’ timeframe and likely to result in a widening of the research-to-practice gap. In addition, those most in need of benefiting from evidence-based interventions (EBIs) may also be the least likely to receive it or receive it as intended [7]. The COVID-19 pandemic has underscored the urgency for implementation science to facilitate equitable responsiveness of existing treatment and prevention efforts for all populations without needing to develop new interventions that will require substantial resources and time to test and disseminate. Thus, we focus on enhancing the rapid response of prevention efforts for high-priority drug use issues, such as opioids or vaping, for diverse populations using existing, widely adopted EBIs and delivery systems. This line of research has the potential to reduce the notable consequences of emerging drugs, including preventable morbidity and mortality among youth.

Widely adopted, universal (i.e., Tier 1) prevention EBIs such as the Michigan Model for Health™ (MMH) offer an unparalleled opportunity to reach large youth populations, including those underserved in other settings such as clinical or community settings [8]. MMH is a Tier 1 or universal health curriculum designed for all students that is used widely across Michigan, 38 US states, and Canada and has shown efficacy in reducing substance use and improving mental health outcomes among students [9–11]. MMH is based on Social Cognitive Theory [12, 13] and the Health Belief Model [14, 15] and is aligned with State- and National Health Education Standards [16]. MMH is skills-focused and addresses cross-cutting risk and protective factors applicable across different types of drug use [17, 18]. Tier 1 EBIs delivered in schools, however, do not always reflect the most current needs of the context and population. Without a rigorous and rapid pathway to respond to emerging drugs and novel routes of administration, EBIs can quickly become obsolete for those most in need. In addition, teachers and other school professionals are rarely given guidance about how to respond effectively when new public health issues arise. A recent systematic review indicated that failing to meet student needs, outdated curriculum materials, lack of updated training, and low staff confidence were key determinants of poor implementation and sustainment of school-based prevention [19]. Previous research on MMH implementation determinants is consistent with this systematic review [20]. Gaps in information related to pressing health concerns such as drug use lead teachers to feel underprepared to address critical, emergent student needs such as new drugs or novel routes of administration and were linked with reduced intervention acceptability and fidelity [20].

Effectively responding to emergent public health issues is a challenging and disparate process for practitioners and researchers. Researchers have often developed new interventions using the traditional, linear translational research approach that takes years and significant resources and often with little exportability or external validity once the initial efficacy trials are completed [21]. Consequently, few community settings benefit from this research [22]. In contrast, practitioners, schools, and other systems that serve youth are often poorly equipped to advance Tier 1 prevention to serve all youth during the initial onset and escalation of crises [23]. Frequently, schools end up concentrating disproportionate effort and resources once an issue has already escalated, as with the vaping and opioid crises, on services among those who have initiated use and/or are at high risk of abuse and addiction, that is, Tier 2 and Tier 3 interventions [3]. Implementation science offers an opportunity to bridge this extant gap between research and practice and more rapidly respond to population needs [24]. While implementation science has made notable advances in many areas, it remains underutilized in facilitating rapid public health responses [25]. For the field to have a more significant “real-world” impact, it needs to be applied more rapidly, iteratively, and work within the time frames of youth-serving systems [25, 26]. After Action Review (AAR), a framework applied in emergency response systems is a promising approach to bridging this gap.

**Fig. 1:**
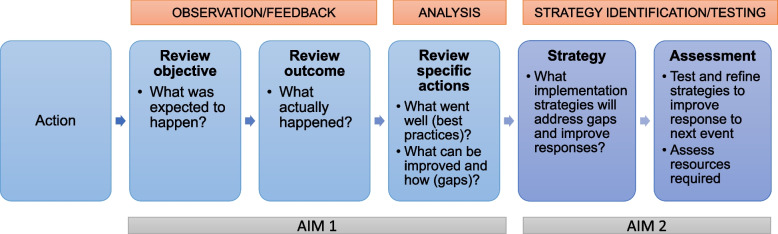
Primary AAR questions and phases, adapted from Villado and Arthur [36]; Salem-Schatz et. al. [32]

AAR is a reflective process that health organizations increasingly use to improve team and system response capacity for emergent public health issues [27]. AAR is a systematic approach to combining the “what” (EBI and delivery system) with the “how” (implementation strategies to improve system responses) to enhance implementation and effectiveness outcomes. Key phases of AAR include: (1) examine current practices, (2) analyze gaps/best practices, and (3) identify and test implementation strategies (e.g., promote adaptability) to improve system responsiveness to the next event (see Fig. 1). AAR has been applied successfully at various systems levels such as a country or health system to strengthen capacity and readiness for responding to emergent public health events [27, 28]. For example, the World Health Organization and other public health entities, as well as healthcare systems, have applied AAR to methodically investigate and enhance their continued and future responses to both emergent and planned events, such as natural disasters and quality improvement initiatives [29–32]. As a recent example, AAR was applied within the U.S. Department of Veterans Affairs (VA) Healthcare system to enhance the responsiveness of residential treatment programs to the COVID-19 pandemic to enable better effective care for veterans experiencing issues with mental health, substance use, and/or homelessness [33]. AAR has also been used to assess and prevent short- and long-term harms of adverse childhood experiences through family-centered treatment for substance use disorders [34] and is recommended as a structured tool for state health departments to use in responding to opioid overdose spikes [35]. AAR offers an opportunity to facilitate effective and timely responses by capitalizing on *existing* system capacity and identifying practical areas for improvement by addressing gaps using implementation strategies. AAR seeks to gain insights from previous responses to identify actions that can be taken in the short term to ensure better preparation for the next event (e.g., emerging drug trends) and identify ways to strengthen and sustain response capabilities (e.g., assessing costs). Despite the expanded use of AAR for urgent public health events, it is underutilized in schools and other primary institutions serving youth.

Researchers suggest that those in most need of benefiting from an EBI may also be the least likely to receive it, or receive it as intended, called the “inverse prevention law [7].” This may be especially true with urgent public health issues. When an EBI improves general population health, it may have little or no impact and possibly exacerbate health inequities for some groups (e.g., low-income populations) who were less likely to be reached or reached effectively [7, 37]. As the COVID-19 pandemic and other public health emergencies have demonstrated, socioeconomically disadvantaged and marginalized populations are most likely to suffer negative consequences disproportionately in the short- and long-term [38]. During the pandemic, we saw exacerbating disparities in mental health and substance use outcomes among marginalized populations who generally have limited access to treatment and prevention services [39]. Thus, it is vital to understand how to improve rapid response efforts to recognize better specific gaps that may contribute to inequities. Schools are well suited to utilizing implementation science to enhance equity as they often have notable experience working with underserved populations [40, 41]. Health equity may be the *central indicator* of success for implementation research but it requires a greater focus on diverse study samples to increase the potential to benefit all populations [40].

Implementation efforts undoubtedly have economic implications. Implementation failure is often due to organizations being unprepared to support effective EBI implementation and/or not understanding what costs they will accrue given their organizational structure [19, 42–44]. A recent systematic review of public health interventions in schools found that costs, including lack of funding, equipment, space, and personnel support, were a central factor influencing the fidelity and sustainment of EBIs [19]. Costs and outcomes (i.e., return on investment) inherently depend on the resources allocated by decision-makers involved and the context in which implementation takes place [45, 46]. Lack of resources is a crucial reason why EBIs are not implemented and sustained in under-resourced schools [47]. Yet, few studies have focused on understanding the costs of implementation efforts from multiple partner perspectives in schools to determine implementation strategy return on investment. To achieve and sustain public health objectives, understanding the value of implementation efforts, that is, obtaining foundational information on implementation costs from multiple perspectives, is central to informing decisions about resource allocation for interventions and their implementation and sustainment [46, 48]. Although schools frequently adapt EBIs, little guidance exists around when and how to assess the economic impact of rapid adaptations [49]. Advancing cost analysis of rapid adaptation and including perspectives most relevant to real-world decision-making can serve as a model for other settings deploying implementation strategies in response to new and emerging public health issues.

### Aims/objectives

The central objective of this study is to systematically design and test a novel set of implementation strategies, RAPD: Rapid Adaptation to Prevent Drug use, to enhance school systems' capacity to address emergent health issues. The implementation strategies will focus on enabling rapid responses using an existing intervention (i.e., MMH) and delivery system to reduce the social, emotional, and economic burden of youth drug use [50].

To accomplish these objectives, this pilot study will pursue the following aims:

#### Primary aims

Aim 1: we will identify gaps and best practices in responding to urgent drug use events using AAR. Organizational or system learning requires assessing previous urgent public health crises to learn from successes and failures and improve future responsiveness [32]. Guided by AAR, we will assess a state’s response to a past event -the vaping crisis- using the MMH health curriculum and delivery system to identify strengths, weaknesses, and areas for improvement using qualitative methods. We will apply these results to systematically design implementation strategies, called RAPD: Rapid Adaptation to Prevent Drug use, to leverage strengths and address gaps. These strategies may include, for example, promoting MMH adaptability, ongoing training, and implementation facilitation or tailored implementation support from practitioners such as school health coordinators.

Aim 2: we will conduct a pilot study to assess the feasibility, acceptability, and appropriateness of the RAPD implementation strategies developed based on Aim 1 findings. The pilot study design will be a cluster randomized trial comparing RAPD to support MMH curriculum delivery versus standard MMH implementation. We use this design to (1) test study procedures and determine whether progressing to a large-scale trial is warranted [51] and (2) assess the feasibility, acceptability, and appropriateness of the proposed strategies to support MMH delivery versus standard implementation [52].

#### Secondary aims

The secondary aims are to (1) assess RAPD costs from multiple partner perspectives and cost-effectiveness versus standard implementation (Aim 3). Other secondary aims include (2) evaluate the fidelity of MMH at the teacher level and key student-level outcomes to assess potential differences between RAPD and standard implementation groups, (3) assess teacher self-efficacy and behavioral capability in delivering MMH with RAPD using mixed methods, and (4) explore potential differences in student outcomes (e.g., drug use risk perception) between RAPD and standard implementation groups.

## Methods

### Aim 1: design and conduct the After Action Review (AAR)

#### Sample and setting

The AAR sample will consist of key implementation partners from across the state, including teachers and other school professionals (e.g., counselors), administrators, health coordinators, Michigan Department of Health and Human Services (MDHHS) staff, and other potential collaborators (e.g., prevention specialists, local public health department staff). We will use purposive sampling to select diverse partners with expertise and direct involvement in school health and addressing substance use issues. Selection criteria will include individuals with demonstrated involvement in implementing or overseeing school health initiatives, specifically around drug use prevention, and those with diverse backgrounds and perspectives. The selection process is driven by Specific Aim 1 of our research, centered on understanding the perceptions, experiences, and challenges encountered in implementing school-based drug use prevention.

#### Procedures

We align the AAR with the World Health Organization’s (WHO) AAR Planning Roadmap and Villado and Arthur’s conceptual framework (see Fig. 1, [51]). Aim 1 focuses on the review component of AAR centered on four key questions: (1) What was expected to happen? (2) What actually happened? (3) What went well (i.e., best practices)? (4) What can be improved (i.e., gaps)? Our steps follow the WHO roadmap, including designing and specifying the AAR approach, conducting the AAR and sharing results, and debriefing and follow-up. The steps are designed to happen rapidly [28], within several months of AAR initiation. AAR is a qualitative review of actions taken in response to a public health event to identify actions that can be taken immediately to ensure better responsiveness to the next event and longer-term actions to strengthen the capabilities of the system in responding to future public health crises [28]. AAR is flexible, adapting to fit the system under review and the event [28]; thus, it is well-suited to assess responses to emerging drug use issues in schools. Finally, we utilize AAR because it explicitly centers on the opportunity to translate experiences from a response into actionable strategies that can be incorporated into the next event.

##### Design the AAR

To structure the scope of the AAR, we use several of WHO’s technical areas of focus for reviewing responses to public health events: surveillance (e.g., early warning, surveillance information, and management), coordination and initial response (e.g., resource mobilization, coordination with other agencies and organizations), and communication and community engagement (e.g., engagement of state, school and community partners). We will include representation of personnel from schools serving low-income and underserved populations and schools serving middle-/upper-income families to assess potential differences in responses. We will collect qualitative data through focus groups, when possible, but also conduct semi-structured interviews to be flexible to the logistical needs of our implementation partners.

##### Prepare and conduct the AAR

We will prepare for conducting the AAR by first obtaining, reviewing, and sharing a common understanding of relevant background information. This involves becoming familiar with each partner group’s baseline standard operating procedures before the vaping epidemic and with any available documentation of MMH's planned updates in response to the crisis. For the AAR sessions, we will develop and refine a list of questions (similar to a focus group interview guide) to determine (1) what was expected, (2) what actually happened, (3) what went well, and how it can be repeated, and (4) what could have been done differently and needs changes.

We will conduct each interview/focus group by first introducing the AAR's agenda, objectives, scope, methodology, and expected outputs to the participants. Each session will follow the protocol suggested by Krueger and Casey [53]. We will facilitate a discussion to identify capacities that existed before the current crisis in terms of plans/policies, resources, and coordinating mechanisms. From the participants ' perspectives, we will attempt to clarify approximate time points at which responses to the crisis occurred, such as initial outreach from MDHHS to health coordinators and health coordinators to teachers about responding to the rapid escalation in vaping. Table 1 includes example questions.

**Table 1:** Questions for the AAR small group sessions (observation and feedback phase of AAR)

Topical category	Example question
**Surveillance**	What went well with rapidly identifying the public health issue (vaping)? How did you receive the information about the escalating public health issue?
**Coordination**	What went well with coordinating efforts across state agencies and organizations involved in adapting MMH to address the vaping epidemic? What resources and supports were available? What resources were needed but not readily available?
**Engagement**	To what extent were various partners engaging in sharing information, resources, and solutions in using the updated MMH? What barriers existed to accessing this information or resources?

### Data analytic approach

#### Rapid qualitative data analysis

We will use rapid turnaround data analysis techniques [54, 55], including structured template data summaries and data matrix displays to compile the data from the small groups. The rapid turnaround analysis approach aims to generate reliable research findings that can be integrated into practice as soon as possible. In addition, researchers have found that rapid turnaround analysis findings generate reliable, timely information similar to other qualitative approaches such as thematic analysis [56]. We will follow the procedure for rapid qualitative analysis described by Koenig et al. [57]. We will summarize each partner interview/discussion using a multistep procedure. First, team members will read through each transcript, noting particularly rich responses, defined as contextually meaningful and detailed responses. Second, we will develop and test a structured template to standardize how discussion content is captured. Specifically, based on the discussion guide, we will identify key topics or themes from the group discussions as broad categories for the summaries. Three team members will test the template with a single discussion, compare summaries, and resolve discrepancies to standardize the summary procedure. Third, after summarizing all the discussions using the template, we will aggregate the data to create a comprehensive matrix. This data matrix summary will systematically compare partner groups [56]. We will identify the key themes within and across topics, including what went well (i.e., best practices) and areas for improvement (i.e., gaps). We will examine potential differences in responses in contexts serving under-resourced populations versus more-resourced populations to elucidate additional gaps contributing to health disparities.

#### Debrief and follow up after the AAR

**Fig. 2:**
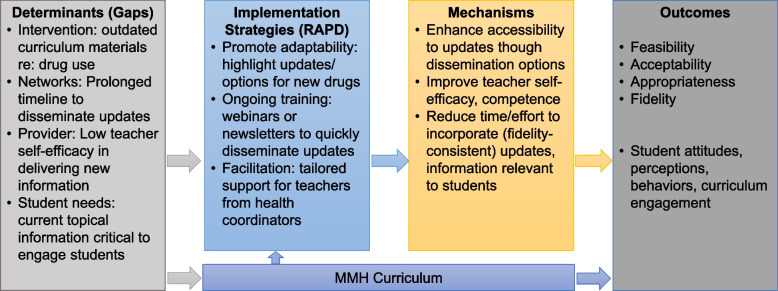
Potential Implementation Research Logic Model for RAPD (Rapid Adaptation to Prevent Drug use); an example set of strategies is listed in the “implementation strategies (RAPD)” box. MMH (Michigan Model for Health) curriculum is the evidence-based intervention being implemented

Debriefing will start with the project team members meeting to discuss and summarize findings. We will then share the findings summary with the AAR participants for validation. Once the participants validate the summary, we will hold a debriefing meeting with the state’s Department of Health and Human Services and School Health Coordinators Association, as well as share information with other partners who regularly collaborate with them, including the Michigan Department of Education and the Michigan Elementary and Middle School Principals Association. We will share the results of the AAR, including the best practices, challenges (i.e., gaps), and follow-up actions that have been identified. This meeting will also be used to discuss the need for additional debriefings, especially with partner groups whose members’ engagement is key to the AAR’s identified follow-up actions. These meetings will culminate in a structured report with concrete recommendations for specific implementation strategies aligned with the identified gaps, including potential equity gaps, to improve the response to the next event using the Implementation Research Logic Model (IRLM; see Fig. 2) [58]. These recommendations will include relevant findings for state agencies and other organizations responding to urgent public health events.

### Aim 2: design and pilot test RAPD deployment

#### Sample and setting

This pilot study, testing the set of implementation strategies designed based on Aim 1 results, will include ten middle schools from regions across the state (urban, rural, suburban) served by participating health coordinators. We anticipate 24 students per school, consistent with the mean number of students per classroom in Michigan [59]. The health coordinators’ service regions include one or more adjacent counties. We will enroll one or more health teachers from each participating school. Students aged 12–14 who are enrolled in the participating teacher’s classroom are eligible to participate in a pre- and post-implementation survey.

#### Sample size

Our proposed sample size includes 10 clusters with an average cluster size of 32 participants, consistent with the range reported in pilot cluster randomized controlled trials (inter-quartile range: 4–16 clusters; cluster size: 14–82) [60]. This sample size is appropriate for a pilot study designed to assess the feasibility and acceptability of implementing the intervention at the school level as primary and to estimate the variability in key implementation outcomes such as fidelity and student outcomes as secondary. This sample size also reflects the available resources for the pilot study while still allowing for meaningful data collection.

The justification for this sample size aligns with best practices for pilot studies, which prioritize achieving sufficient precision for key implementation parameters rather than powering analyses for statistical significance [52]. Given the exploratory nature of pilot studies, formal power calculations are not required, as the primary aim is to assess feasibility, variability, and implementation processes [61]. To ensure that our pilot sample will allow us to draw reasonable conclusions for the parameter estimates in a larger study, we conducted an AIPE (Accuracy in Parameter Estimation) analysis. We found that across 1000 simulations, the average 95% confidence interval widths for the student outcomes were 0.18 standardized units, and 0.85 for school-level variability. The focus on AIPE is more central to our aim in this pilot study than statistical power, especially for the school-level effects. Thus, our proposed sample size reflects a balance between the pragmatic constraints of a pilot trial and the need to collect adequate data to inform the design and conduct of subsequent larger-scale effectiveness trials.

#### Eligibility and randomization

We will focus on schools that could most benefit from RAPD by employing the following screening criteria: schools must include seventh-grade education level; health teachers must report delivering less than 80% of the MMH curriculum (the state-identified fidelity standard) and/or report two or more barriers to MMH implementation (see “Measures”). These criteria are adapted from a previous school-based study and informed by study partners and the study team's previous research on MMH implementation [62, 63]. Five of the matched sites will be randomized to the RAPD and five to standard implementation: the MMH curriculum manual, training, and technical assistance. Research team members will use the GenRandGroups function within the DescTools package (version 0.99.54) in R. The randomization procedure is designed to pilot methods to ensure balanced participant allocation across experimental conditions. Schools will be the randomization unit, with matching criteria for assigning schools to treatment groups involving factors such as school size, the percentage of students eligible for free/reduced lunch, and the health coordinator serving the school. Participants (schools) were not masked as is not practical or desired in this implementation study, which focuses on improving the speed, quality, and quantity of intervention delivery through different strategies, acknowledging the influence of participants' awareness on observable outcomes. The health coordinators have successfully recruited schools for previous similar studies [63, 64]. All standard implementation sites will receive RAPD following the trial portion of the study. We focus on middle school because of the heightened risk for drug use initiation during this developmental period [65].

### Procedures

#### RAPD implementation strategy design

We will use the Implementation Research Logic Model (IRLM) to organize and synthesize the gaps identified in Aim 1 into actionable implementation strategies to address these gaps. This process will support the rigor and reproducibility of this research for broad application when engaging in rapid responses. While the findings from Aim 1 will ultimately shape the implementation strategy specification for the pilot trial, collectively referred to as RAPD, we provide an example of anticipated key determinants (i.e., gaps; see Fig. 2) based on our previous and preliminary research [20, 66], existing research [19, 67], and other work focused on Tier 1 substance use prevention [63, 68]. Our logic model specifies determinant relationships with implementation strategies, mechanisms of action, and anticipated outcomes [58]. We will use the Consolidated Framework for Implementation Research (CFIR) to identify and organize the “gaps” to be targeted for implementation strategies. CFIR is a comprehensive determinant framework that includes domains across socioecological levels (e.g., intervention, inner and outer context, provider characteristics) and whose overarching aim is to understand and identify central factors, that is, gaps and best practices, that influence intervention delivery in a given setting [69, 70].

As implementation is inherently complex, researchers and practitioners are challenged to identify the consistent impact of implementation strategies to mitigate barriers to effective intervention delivery [71]. Systematic processes for matching gaps and implementation strategies and assessing their proposed mechanisms of action are needed. Implementation science researchers have created tools, such as the Consolidated Framework for Implementation Research (CFIR)-implementation strategy matching tool online to support researchers and practitioners in selecting suitable strategies [72]. We will use the SISTER: School Implementation Strategies Translating ERIC (Expert Recommendations for Implementing Change) Resources taxonomy to specify implementation strategies [56]. We will also consider and assess possible mechanisms of action, that is, processes through which implementation strategies operate to influence outcomes [73]. Examples of possible strategies include promoting intervention adaptability, providing ongoing training, and implementation facilitation.

#### Promoting EBI adaptability

To promote adaptability in drug use prevention programs, we will use fidelity-consistent adaptations that meet the population's specific needs (e.g., new drugs) while preserving the core elements of evidence-based interventions [74]. The interventions' core functions align with their intended outcomes and purpose [73]. For instance, we systematically incorporate up-to-date drug use prevention information, like the vaping example from Aim 1, while maintaining the essential components of the intervention (MMH core functions). Thus, we will collaborate with state agencies and community partners to facilitate these updates and enhance adaptability. We will share information from ongoing drug use-related efforts and swiftly integrate available options, information, and resources into the MMH-the form elements. Form elements can be tailored to the setting [75]. We will provide briefs on new policies, expert preliminary guidelines, and supplementary resources for the curriculum and families.

Recognizing the diverse needs of schools, especially those serving economically disadvantaged and marginalized students, we will offer tailored options for curriculum adaptation. These include information sensitive to families at higher risk of substance use disorders or adversity and activities with additional support and evaluation to foster skill development. This flexible and targeted approach enables us to address emergent drug use issues that complement the existing MMH curriculum.

#### Ongoing training

Researchers have found that the uptake, fidelity, and sustainment of EBIs can be markedly improved by providing ongoing training; this is especially useful when tailored to the local needs and context, including the unique needs of low-resource environments [75]. Training is a valuable strategy that can be used to adopt and sustain innovations effectively [76]. Teachers faced with providing content outside their expertise, as with new drugs, may be less likely to deliver the content with fidelity, if at all [77, 78]. Training can improve behavioral capability by building knowledge, skills, and self-efficacy; this will build mastery for new content delivery, improving fidelity [71, 79]. Health coordinators are well suited to providing ongoing teacher training as this is within their current scope of practice. Training can also promote implementation and sustainment by enhancing staff’s EBI enthusiasm, vital to promoting intervention fidelity [77, 78, 80].

#### Implementation facilitation

 Is a crucial aspect of successful implementation, supported by research indicating that having a community-based expert and support person, such as a school health coordinator, significantly increases the likelihood of positive outcomes [76]. In the context of our intervention, facilitation serves as a flexible strategy tailored to address barriers related to the intervention, context, recipients (students), or providers. Based on the integrated Promoting Action on Research Implementation in Health Services (iPARIHS) framework, facilitation involves providing individualized assistance for intervention delivery [81]. This approach includes interactive problem-solving and ongoing collaboration to overcome barriers tailored to the provider and contextual needs. Tailored support is vital to effectively reach underserved populations, including organizations serving youth at higher risk of substance use disorders, and to achieve desired public health outcomes [82]. Although facilitation has predominantly been deployed in clinical settings [83, 84], preliminary evidence from a recent, large-scale, school-based implementation study suggests that facilitation also positively impacts school EBI adoption [85]. Therefore, facilitation will likely enhance the acceptability of our MMH program and boost teacher self-efficacy in delivering the intervention.

### Study design

**Fig. 3:**
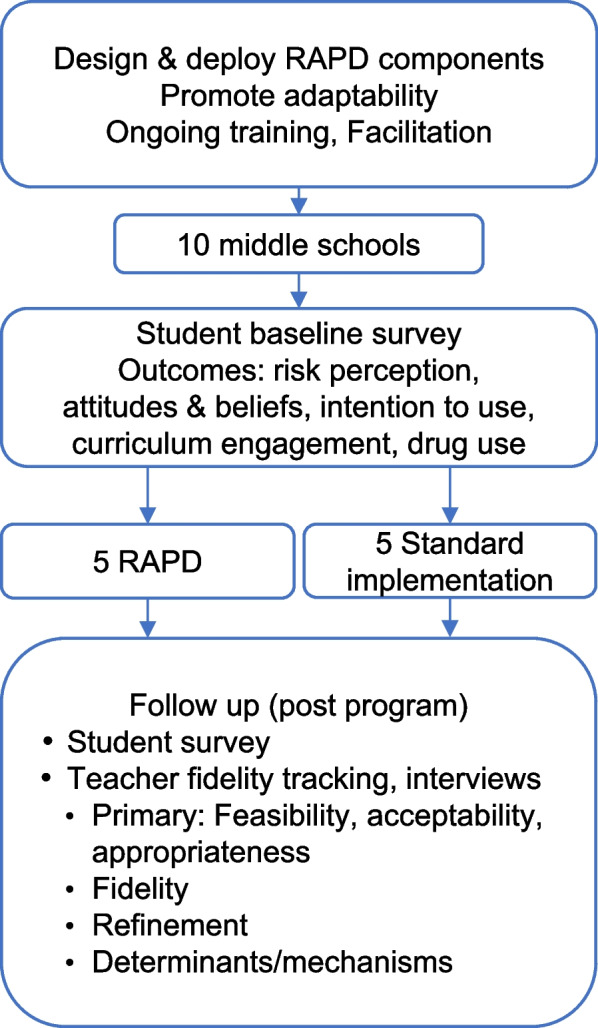
Aim 2 study design

The pilot study will use a mixed method, hybrid effectiveness-implementation, 2-group cluster-randomized trial (CRT) design to investigate deploying RAPD for delivering MMH (Fig. 3). Specifically, the study uses a hybrid type 2 approach, which includes evaluating potential implementation strategy effects on provider practices and exploring participant outcomes [86, 87]. Standard implementation consists of the MMH digital curriculum, standard MMH training, and as-needed technical assistance from the health coordinators. RAPD will augment standard implementation with additional strategies based on Aim 1 results. See Fig. 2 for possible RAPD strategies and Fig. 3 for pilot study design.

### Pilot study procedures

Per institutional IRB approval and to ensure ethical compliance, members of the study team and school health coordinators, including the study manager and Principal Investigator (PI), will disseminate study information to teachers, administrators, and parents at participating schools. Consistent with similar surveys in schools, we will provide parents with an opt-out option, and participant assent will be required before survey access. Students, then, will be allowed to participate in the study unless their parents explicitly choose to opt them out. Students will complete a self-administered survey with a unique identifier not linked to school or personal information. We will administer the survey through a secure online server to protect data confidentiality. As the MMH is incorporated into the school curriculum, students will complete the surveys during their health class before MMH delivery and upon course completion. For students without parental consent or those who choose not to assent, an alternative activity, such as a worksheet, will be provided to ensure their inclusion in school activities. Based on a 75% response rate, as observed in other community studies [88], we expect approximately 450 students to participate in the survey. As a study consistent with educational practices and existing school-based survey administration, there are few risks associated with participation; the primary adverse event of concern is potentially upsetting survey questions. Students who feel uncomfortable or upset by survey questions are permitted to skip questions or end participation at any time and are encouraged to report their concerns to their classroom teacher, which will then be shared with the study team. The researchers will report any adverse events to the institutional IRB.

### Measures

#### Primary outcomes: feasibility, acceptability, and appropriateness

We will assess the feasibility, acceptability, and appropriateness of deploying the RAPD implementation strategies using a mixed methods approach: (1) we will conduct semi-structured interviews with the pilot study middle school health teachers at three different time points: pre-implementation, during implementation, and post-implementation of the MMH program. We will conduct these interviews via videoconferencing and record and transcribe them verbatim. Our assessment will focus on the compatibility of delivering the MMH curriculum with RAPD in addressing key barriers (e.g., intervention's capacity to address new drugs) and the perceived fit of MMH within the context regarding appropriateness [86]. (2) We will administer a survey assessing feasibility, acceptability, and appropriateness using items from Weiner et al. [89] during the post-implementation interview. Each construct will use a 5-point Likert scale and consist of four items. For example, participants will rate their agreement on statements such as “RAPD is appealing” and “RAPD seems suitable.” These quantitative measures will provide valuable data to complement the insights gained from the qualitative data.

### Secondary outcomes

#### Fidelity: dose delivered, adherence

We will utilize an existing MMH fidelity tracking form administered at the end of each unit currently used by health teachers to gather data on curriculum use. We will assess the dose delivered by summing the lessons covered during the drug use prevention units (17 lessons). We will assess adherence by examining reports of key functional content areas addressed in the drug use prevention units, such as evaluating the problem, identifying reliable sources of information, and practicing key prevention skills (e.g., refusal skills). We will also use the tracking form to assess adaptations based on the Framework for Reporting Adaptations and Modifications-Enhanced (FRAME), e.g., added content, removed content [90].

#### Teacher self-efficacy and behavioral capability

We will adapt the GSE-6 (General Self-Efficacy Scale) [91] for the context and tailor behavioral capability items using the CDC’s characteristics of effective health education [92] and assess these constructs in semi-structured interviews.

#### Student outcomes

Secondary outcomes at the student level are summarized in Table 2. Student surveys will be administered at baseline (the beginning of the semester or trimester), when the student is in health class, and after delivery of the drug use prevention units (post-test). During the school year, participating teachers will administer the pre-/post surveys for each cohort of students if they teach health for more than one term/year.

**Table 2:** Secondary study measures student level

Student-level outcomes			
**Construct (# items)**	**Scaling**	**Timing**	**Source**
**Effectiveness: risk perception**			
Occasional use [drug] (1)	1 = no risk – 4 = great risk	Pre-/post-implementation	MTF^a^ [1]
Regular use [drug] (1)	1 = no risk – 4 = great risk	Pre-/post-implementation	MTF^a^ [1]
**Implementation outcomes: fidelity engagement**			
Satisfaction (4)	5-point Likert	Post-implementation	Giles et al. [93] adapted
Key skills: assertive communication, refusal skills, decision-making (9)	5-point Likert	Post-implementation	National Health Educ Standards [16], MMH [94]
**Effectiveness: drug use behaviors**			
Drug use (vaping, cannabis, alcohol, prescription medications) (16)	1 = none 7 = 40 or more times	Pre-/post-implementation	MTF^a^ adapted [1]
**Disparities: measures of socioeconomic disadvantage**			
Economic disadvantage: food insecurity (9), free/reduced lunch (1)	Insecurity: 1 = a lot to 3 = never; eligibility: yes, no	Pre-implementation	US Food Security Survey Module [95]

^a^Monitoring the Future

### Data analytic approach: primary outcomes

#### Qualitative data

We will use an inductive/deductive thematic analytical approach outlined by Hsieh and Shannon for interview transcripts [96]. First, the study staff and the PI will review the transcript material to develop a broad understanding of the content related to the project’s specific aims. We then will identify topics of discussion and observation. During this and subsequent steps, we will document initial impressions of topics and themes and their relationships to each other to define the boundaries of specific codes [56]. Second, project team members will independently code the interview empirical material to condense the data into analyzable units. Segments of text ranging from a phrase to several paragraphs will be assigned codes based on a priori or emergent themes, also known as open coding [97]. We will assign codes to describe connections between categories and between categories and subcategories, that is, axial coding [97]. We will match lists of codes developed by each investigator and integrate them into a single codebook. Third, two staff will independently code text. We will resolve disagreements through discussion between investigators to create an enhanced definition of codes, resulting in a final codebook. Using this codebook, two study team members will separately review transcripts to determine the level of agreement in the codes applied. A level of agreement greater than 80% indicates good reliability in qualitative research [98]. Fourth, based on these codes, we will use the computer program QSR NVivo [99] to generate a series of categories connecting text segments grouped into separate “nodes.” We will use these nodes and node structures to further the process of axial coding to examine the association between different a priori and emergent categories. Fifth, by continually comparing these categories, we will condense the distinct categories into broad themes.

#### Quantitative data

We will characterize the primary outcomes of feasibility, acceptability, and appropriateness of the RAPD strategies using means and 95% confidence intervals, medians, and interquartile ranges or counts and percentages as appropriate.

#### Mixed method data integration

To integrate both quantitative and qualitative data effectively, we will follow the steps outlined by Creswell and Plano Clark [100]. These steps will enable us to derive meta-inferences, that is, insight beyond each data strand alone when combining data from both sources [96]. We will utilize a spiraled comparison approach, engaging in a continuous iterative process of analyzing and comparing the quantitative and qualitative data strands. This iterative analysis will involve moving back and forth between the two data types, cyclically considering their respective results to merge and enrich our findings [100]. This iterative process will help us identify areas of convergence, divergence, and expansion, ultimately allowing us to develop interpretations or meta-inferences [100] that address our research questions concerning the feasibility, acceptability, and appropriateness of RAPD. To ensure the validity of our interpretations, we will utilize other data sources (e.g., notes from health coordinator facilitation meetings) to develop a comprehensive understanding of the phenomena under investigation, testing the robustness of our findings [101]. Additionally, we will engage in member checking, involving partners, including MDHHS and Michigan School Health Coordinators Association, to review our results following data analysis. This collaborative process will help us validate our interpretations and ensure alignment with the perspectives and experiences of key partners. Any discrepancies that arise during the analysis process will be addressed through methods such as re-analysis of the existing data or, if necessary, by collecting additional data [100]. This approach will allow us to resolve inconsistencies and enhance our study’s overall rigor and validity.

Guided by Pearson et al. [52], we will employ a mixedmethod approach using quantitative and qualitative criteria, along with a “traffic light” system (red, yellow, green), to assess a priori progression to a larger-scale trial. For Aim 2 feasibility outcomes (recruitment, retention, data collection procedures and measures, and feasibility), “green” indicates meeting all pre-defined feasibility targets, “yellow” signifies meeting some targets with potential modifications, and “red” indicates unmet targets requiring substantial revisions.

### Data analysis: secondary outcomes

#### Teacher data

Consistent with Aim 2 primary outcomes, we will analyze the teacher-focused secondary quantitative outcomes (e.g., fidelity) using means and 95% confidence intervals, medians, and interquartile ranges or counts and percentages as appropriate.

#### Student data

Aim 2 analyses of student survey data will focus on estimates of effect size, variance, implications for statistical power, and exploring potential differences between the RAPD implementation strategy and standard implementation on student perceptions of risk, attitudes, behaviors, and MMH engagement. Given the study design, we will utilize mixed-factorial statistical analyses to characterize the magnitude of differences of the pre-/post changes in measures of risk, attitudes, beliefs, and other student-level outcomes. Each model will utilize random Y-intercepts to accommodate the repeated observations within student and school, and fixed-effects parameters comparing time (pre-/post changes), group (RAPD, standard implementation difference), sex/gender, socioeconomic status, and their interaction (as appropriate given sample size constraints). We will employ mixed-factorial regression models for outcomes assumed to follow a normal distribution, logistic regression for dichotomous outcomes, or ordinal regression for outcomes ordinal in scale. The analyses will use Satterthwaite corrections for degrees of freedom.

#### Missing data

Before data collection, the team will meet to discuss plausible reasons for missing data and determine items related to those reasons to include in the quantitative survey as auxiliary variables (i.e., variables that might lead to non-random patterns of missingness but are not of interest as part of the main hypotheses) to increase the likelihood of missing data being due to MAR (missing at random) and MCAR (missing completely at random) processes. In addition to using added items to inform future handling of missingness (i.e., multiple imputation, FIML (full information maximum likelihood)), we will calculate informative principal components [102] so our handling of missing data is informed by all available data, including multivariate interactions, without adding undue burden on model estimation stemming from a large number of variables.

### Aim 3 (exploratory aim): assess the costs and benefits of RAPD from multiple partner perspectives

#### Study design

**Fig. 4:**
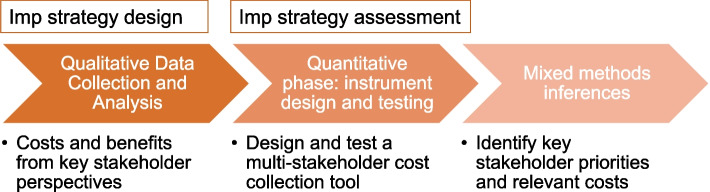
Aim 3 exploratory sequential mixed methods design

We use an exploratory sequential mixed methods design to draw inferences about key costs and benefits that would not be feasible with one method alone (see Fig. 4) [45]. Costs are inherently dependent on the partners and decision-makers involved and the implementation context [45, 46]. We will employ this design to obtain information about costs and priorities from essential perspectives central to MMH implementation and sustainment and most relevant to the context that can inform future financing and issues of funding stability of implementation strategies, consistent with previous research [103]. Because the key costs and benefits across multiple partner perspectives are largely unknown, we use this study design to develop a costing tool tailored to the setting, making it more likely that the tool will be helpful to the participants being interviewed. This approach will advance pragmatic costing tools that account for multiple perspectives and understand implementation costs and benefits across perspectives that can support sustainment.

#### Procedures

During Aim 1 focus groups, we will include questions on perceptions of costs, benefits, and priorities in implementing and sustaining the rapid responses to public health events from each partner’s perspective. Focus groups are useful when exploring perceptions of products, such as the MMH curriculum, and services, such as training or support, and their associated costs, and therefore suitable for this aim. Groups will be asked open-ended questions using a semi-structured interview guide to identify their perceptions of the costs and benefits of MMH implementation and asked to prioritize them. Focus groups will convene virtually, and we will record meetings and transcribed using an online conferencing platform. Qualitative data collection and analysis will be as described in Aim 1. Quantitative: we will develop a costing tool for partners considering implementation to estimate the net costs of using RAPD versus standard implementation through an activity-based costing (ABC) approach, considering multiple perspectives. See Table 3 for potential costs and priorities across partner groups. Consistent with emerging recommendations in the field, we will focus on costs required for replication versus research or development costs [104]. Identifying key implementation costs based on the qualitative data phase will provide practical information for partners regarding resources required for EBI delivery with high fidelity and potential advantages, from an economic perspective, of using RAPD versus standard implementation. Further, identifying costs, benefits, and priorities will also elucidate areas of synergy and conflict across perspectives as a first step in resolving potential conflicts.

**Table 3:** Example key cost considerations for different interested parties, adapted from clinical contexts, Eisman et al. [44]

Interested party perspective	Key priorities	Example of pre-implementation costs	Example of implementation costs	Example sustainability costs
State agencies	Maximize public health impact, est. effective practices	Costs for MMH updates, review, formatting, promotion	Developing and executing health coord trainings	Review and revisions, updating assessments, committee meetings
System (i.e., regional service agency)	Stay within budget; align with the mission (education)	Capital expenses; technology upgrades,	Time costs for logistics, dissemination, training, facilitation	Time costs for updating priorities, alignment with state policies
Organization (i.e., school)	Stay within budget; meet benchmarks (education)	Substitute teacher costs, time, and space costs for updated training	Opportunity costs of time spent deploying RAPD	Costs to maintain quality implementation
Provider team: administrative staff	Effective allocation of staff/resources, meet youth/family needs	Costs of promotion and onboarding	Opportunity costs of time spent on RAPD versus other initiatives	Retraining costs; cost audit and feedback
Provider team: teachers, professionals	Improve workflow; competing demands; relative benefit	Opportunity costs for training, logistics, and implementation prep	Time required to review, incorporate updates into lessons	Opportunity costs for ongoing activities, training

### Measures

#### Qualitative

We will design a focus group guide consisting of three primary questions. Question 1 will ask participants to discuss their perceptions and experiences with the costs and resources associated with MMH implementation and who bears those costs. Question 2 will ask about resource barriers to effective implementation. Question 3 will ask participants to discuss their priorities of costs and benefits related to the MMH implementation. Using Krueger and Casey’s protocol [53], we will encourage groups to deeply explore and expand on their responses.

#### Cost identification and tracking tool

We will identify resources needed for activities during the implementation strategy deployment process, from exploration to sustainability, consistent with other implementation costing tools [104] that map activities across implementation phases based on the results from the qualitative analysis. We will prospectively collect cost data and pilot-test our costing tool. The partner groups, including health coordinators, MDHHS staff, school admin, and teachers, will track their time, other relevant costs, activities/tasks, or benefits as part of RAPD deployment. These tasks may include developing an implementation plan, training, linking to resources for curriculum updates, and technical assistance.

### Data analysis

#### Qualitative

As part of the Aim 1 data analysis, the research team will examine responses to each cost/resource-related question within each partner group, applying a code to capture similar responses. We will organize codes into themes and analyze themes to identify the frequency, extensiveness, intensity, specificity, internal consistency, and participant perception of importance for each theme. We will organize results side-by-side in tables to allow for comparison across partner groups. We will use the analysis of themes (frequency, extensiveness, intensity, consistency) to develop items for the costing tool that captures the themes in the qualitative analysis. We will use a modified Delphi technique to refine the tool and incorporate expert opinion from community partners (i.e., the health coordinators and school personnel), that is, practice-based evidence [105]; upon the first iteration of the tool, we will share it with key partners to provide additional input in advance of cost data collection.

#### Cost

We will calculate costs based on time spent by hourly wage plus fringe rates for staff using Bureau of Labor Statistics data. All costs will be adjusted to current US dollars using the Consumer Price Index [106]. We will estimate other non-labor costs based on study data (e.g., materials). We will report summary statistics, including mean costs with 95% confidence intervals.

## Discussion

### Expected outcomes

We expect Aim 1 outcomes will contribute toward identifying critical gaps in meeting population needs regarding emerging drugs and addressing health disparities in the impact of drug use in under-resourced communities. We expect Aim 2 outcomes will contribute toward designing and testing strategies for drug use prevention in real-world settings and finding ways to reduce health disparities. This includes systematically designing RAPD, that can be applied to other drugs, interventions, and urgent public health issues. We also expect that Aim 2 will result in a feasible, acceptable, and appropriate set of strategies to enhance MMH fidelity and effectiveness and provide an infrastructure for future emerging drugs. This aim will also collect data regarding deploying RAPD in schools serving low-income students, which will be critical to further refinement to enhance equity in preparation for a future large-scale type 3 hybrid trial. We also expect that students in schools receiving RAPD will report increased risk perception and higher levels of student engagement compared to those in standard implementation schools. This mixed methods research will support a subsequent, large-scale trial to address urgent and emerging drug issues among youth and address disparities in system responses. For Aim 3, we expect to develop an implementation strategy costing tool that captures vital interested party priorities and relevant costs related to adaptation to inform a comparative economic evaluation for the larger implementation trial.

### Limitations and future directions

This study is limited to a single state (Michigan), which may hinder generalizability. The pilot study, centered on pragmatic and real-world considerations, has notable potential to offer valuable insights that contribute to the existing literature and future directions for rapid responses to emerging drugs. Additionally, researchers acknowledge the dearth of data to inform optimal sample sizes for pilot cluster randomized trials; thus, while our proposed sample is consistent with other studies, the sample size may pose a risk of bias in results [60, 107]. Also, although AAR has been used to systematically learn from and improve responses to various crises, there is limited prior research on its applicability specifically to enhancing drug use prevention interventions, which this work aims to contribute directly.

This research will advance the application of implementation science in attaining access, cost, and equity of EBIs directed at reducing drug use among adolescents. Through facilitating rapid responses using existing interventions, results from this study will fill a critical gap in our ability to stem the rapid onset and escalation of drugs among youth. This research will provide the foundation for a large-scale hybrid trial to investigate the effectiveness and cost-effectiveness of RAPD and enhance the public health impact of existing EBIs in schools that may be used as a model for other settings. This will also advance the application of implementation science to enhance health equity and ensure all youth benefit from school EBIs. The next step in this research program will be to test RAPD on a larger scale and over a longer duration. We anticipate proposing a large-scale cluster randomized trial with middle schools throughout Michigan and across other states, as many states outside of Michigan use MMH, to assess the long-term impact of RAPD on intervention fidelity and youth drug use-related outcomes. We will also evaluate the costs and cost-effectiveness of RAPD versus standard implementation. Understanding cost and benefit implications and perceived value from various perspectives, from teachers to administration to the larger school and educational system, and what implementation cost information is most beneficial for each group is critical in moving the economic evaluation of implementation research forward [44]. This research will also be a foundation for advancing implementation strategies for rapid responsiveness and scaling up these strategies to achieve equitable public health impact of EBIs. The various processes utilized in this research will identify key costs and benefits from multiple partner perspectives. This knowledge will be critical to informing economic evaluation to guide decisions about resource allocation. This will also inform partners what successful implementation of strategy adoption and sustainment will cost them.

We expect this research to contribute to advances in systematically applying implementation science to rapidly, equitably, and cost-effectively address emergent public health issues within and beyond youth drug use and school-based prevention.

## Data Availability

The data generated and analyzed during this study will be made available to the corresponding authors upon reasonable request, consistent with NIH guidelines.

## Abbreviations

EBI: Evidence-based intervention
MMH: Michigan Model for Health
AAR: After Action Review
RAPD: Rapid Adaption to Prevent Drug Use
MDHHS: Michigan Department of Health and Human Services
IRLM: Implementation Research Logic Model
CFIR: Consolidated Framework for Implementation Research
ERIC: Expert Recommendations for Implementing Change
SISTER: School Implementation Strategies Translating ERIC Resources
MTF: Monitoring the Future
